# Supplementary material for: Genetic data of museum specimens allow for inferring evolutionary history of the cosmopolitan genus Sirthenea (Heteroptera: Reduviidae)
Source: PeerJ. 2019 Apr 10;7:e6640. doi: 10.7717/peerj.6640 (PMC6462186; doi:10.7717/peerj.6640)
Supplement: Supplemental Information 1 [file peerj-07-6640-s001.docx]

**Detailed list of specimens examined for the morphological data matrix with information about localities of collection**

1. ***Sirthenea* *africana* Distant, 1903:**

**Botswana**: Male: BOTSWANA (B 15) / R. Thamalakane, / 7 mls. NE. Maun / 20.iv.1972 [printed]; at / light [printed]; Southern / African Exp. / B.M. 1972-1 [printed] (NHMUK). **Democratic Republic of Congo**: Male: Holotypus [handwritten on orange label]; Musée du Congo [printed] / Mufungura / 15-X-1912 / (Dr. J. Bequaert) [handwritten]; R.Det . / F / 2453 [printed]; Sirthenea / bequaerti / Sch. / Type [handwritten] (RMCA). Male: Holotypus [printed on orange label; Musée du Congo [printed] / Bas Uele: Bambesa]: / 1938 / (Leontovitch) [handwritten] R. Det / 2453 [printed]; Sirthenea / leontovitchi / Sch. / Type [handwritten] (RMCA). Female: Holotypus; Musée du Congo / Boma [printed] / 12-VII-1920 [partially handwritten] / Dr H. Schouteden [printed]; R. Dét [printed] / H [handwritten] / 2453; Sirthenea / rapax var. / concolor Sch. / Type [handwritten] (RMCA). Female: Musée du Congo / Région d’Abok [printed] / -1935 [printed]; M & Me Ch. Scops [printed]; Sirthenea / leontovitchi / Scht [handwritten] (RMCA). Male: Musée du Congo / Bamabnia [printed] / -1934 [printed]; R. Fr. Longinus [printed]; R. Det / 6968 [printed] / B [handwritten] (RMCA). Female: Musée du Congo / Mahagi-Niarembe [printed] / IX-1935 [printed]; Ch. Scops [printed]; Sirthenea / leontovitchi / Scht / Det: Schuteden [handwritten] (RMCA). Female: Musée du Congo / Mahagi-Niarembe [printed] / -1935 [printed]; Ch. Scops [printed]; Sirthenea / leontovitchi / Scht / Det: Schuteden [handwritten] (RMCA). Male: Musée Du Congo / Lulua: Tshibalaka / X-1933 / F.G. Overlaet [printed]; Sirthenea / rodhaini Sch [handwritten] (RMCA). Male: Congo Belge P.N.A / 18-VIII-1953 / Vanschuytbroeck / V. Hendrickx 5335 [printed]; Massif Ruvenzori / Kalonge, 1900 m / Riv. Kamusonge, / aff. Butahu [printed]; Coll. Mus. Tervuren [printed]; Sirthenea / bequaerti / Schout. [handwritten] / A. Villiers det 195 [printed] (RMCA). Female: Coll. Mus. Tervuren [printed]; Kiva N: Mutsora / 2.II.1953 / Mission de Witte [handwritten] (RMCA). Male: Musée du Congo / Flandria [printed] / 15-III-1932 [handwritten, partially printed]; R.P. Hulstaert [printed]; R. Det / 6968 [printed] / B [handwritten] (RMCA). Male: Coll. Mus. Tervuren / Tschuapa: Bamanya / 1968 / Rev. P. Hulstaert [printed] (RMCA). Male: Musée du Congo / Equateur: Flandria [printed] / X-1931 [handwritten, partially printed]; R.P. Hulstaert [printed]; R. Det / 6968 [printed] / B [handwritten] (RMCA). **Ethiopia**: Female: ETHIOPIA / Illubabor Prov. / Gambela [printed] AT UV LIGHT [handwritten] / 15-11-1972 [partially handwritten] / G.B.White / B.M. 1974-85 [printed] (NHMUK). 2Males: ETHIOPIA / Illubabor Prov. / Gambela [printed] UV LIGHT [handwritten] / 15-11-1972 [partially handwritten] / G.B.White / B.M. 1974-85 [printed] (NHMUK). **Gabon**: Female: Gabon 1935 II [printed] / Libreville [handwritten] / Coll. J. Primot [printed]; Sirth enea / rapax / Horv. [handwritten]; det. R.J.Izzadr 1937 [printed, date partially handwritten] (NHMUK). **Ghana**: Female: Ghana: Ashanti region / Kwadaso, 259 m / N 6º 42’ - W 1º 39’ / Dr. S. Endrody-Younga [printed]; Nr. 341 / mixed light / 28.IV.1969 [printed]; Sirthenea rapax / Horváth, 1909 [handwritten] / det. Rédei D., 2003. [printed, date partially handwritten]; Hung. Nat. Hist. Mus. / Budapest / Coll. Hemiptera (HNHM). Female: Ghana: Ashanti region / Kwadaso / 259 m, N 6 55 - W 1 39 / Dr. S. Endrody-Younga [printed]; Nr. 367 - light trap / on field / 2.VI.1969 [printed]; Sirthenea rapax / Horváth, 1909 [handwritten] / det. Rédei D., 2003. [printed, date partially handwritten]; Hung. Nat. Hist. Mus. / Budapest / Coll. Hemiptera (HNHM). Male: Ghana: Ashanti region / Kwadaso, 259 m / N 6º 42’ - W 1º 39’ / Dr. S. Endrody-Younga [printed]; Nr. 341 / mixed light / 28.IV.1969 [printed]; Sirthenea rapax / Horváth, 1909 [handwritten] / det. Rédei D., 2003. [printed, date partially handwritten]; Hung. Nat. Hist. Mus. / Budapest / Coll. Hemiptera (HNHM). Male: Ghana: Ashanti region / Kwadaso / 320 m, N 6 42 - W 1 39 / Dr. S. Endrody-Younga [printed]; Nr. 366 - light trap / on field, UV light / 26.V.1969 [printed]; Sirthenea rapax / Horváth, 1909 [handwritten] / det. Rédei D., 2003. [printed, date partially handwritten]; Hung. Nat. Hist. Mus. / Budapest / Coll. Hemiptera (HNHM). Male: Ghana: Ashanti region / Kwadaso / 259 m, N 6 55 - W 1 39 / Dr. S. Endrody-Younga [printed]; Nr. 366 - light trap / on field, UV light / 26.V.1969 [printed]; Sirthenea rapax / Horváth, 1909 [handwritten] / det. Rédei D., 2003. [printed, date partially handwritten]; Hung. Nat. Hist. Mus. / Budapest / Coll. Hemiptera (HNHM). Male: Ghana: Ashanti region / Kumasi, Rhiasu / 350 m, N 6 43 - W 1º 39’ / Dr. S. Endrody-Younga [printed]; Nr. 300 / UV light / 7.II.1968 [printed]; Sirthenea rapax / Horváth, 1909 [handwritten] / det. Rédei D., 2003. [printed, date partially handwritten]; Hung. Nat. Hist. Mus. / Budapest / Coll. Hemiptera (HNHM). Male: Ghana: Ashanti region / Kwadaso / 320 m, N 6 42 - W 1 39 / Dr. S. Endrody-Younga [printed]; Nr. 327 / mixed light / 18.III.1969 [printed]; Sirthenea rapax / Horváth, 1909 [handwritten] / det. Rédei D., 2003. [printed, date partially handwritten]; Hung. Nat. Hist. Mus. / Budapest / Coll. Hemiptera (HNHM). **Malawi**: Female: Mt. Mlanje / Nyassaland [printed] / 21.IX 1913 [partially handwritten] / S.A.Neave. [printed]; Pres. by / Imp. Eur. Ent. / Brit. Mus. 1927-85 [printed] (NHMUK). Female: Mt. Mlanje / Nyassaland [printed] / 29.VIII 1913 [partially handwritten] / S.A.Neave. [printed]; Pres. by / Imp. Eur. Ent. / Brit. Mus. 1927-85 [printed] (NHMUK). Male: Valley of / Lowes / Shide [handwritten] / Nyassaland [printed] / 22. VII 1913. [partially handwritten] / S.A. Neave [printed]; Pres. by / Imp. Eur. Ent. / Brit. Mus. 1927-85 [printed] (NHMUK). **Mozambique**: Female: Museum Paris / Moçambique / Prov. De Gorongora / Tendo de Sungoue (40 m D’Alt) / C. Vasse 1907 [printed on green label] (MNHN). **Nigeria**: Female: N. Nigeria: / Zaria, Samaru. / 17.XI. 1967. [printed, date partially handwritten]; J.C.Deeming / m.v. trap [printed]; Sirthenea / rapax Horv. / var. concolor / Schout. [handwritten] / Det.JC.Deeming 1967 [printed, date partially handwritten] (NHMUK). Male: Ibadan Nigeria / At Light Trap / D/C:I:II:66 [handwritten]; 19 [handwritten]; Sirthenea / rapax Horv. [handwritten] / M.S.K. Ghauri, det.1968 [printed, date partially handwritten]; Pres by Com Inst Ent / B M 1958 26 [printed]; C.I.E. Coll [printed] / A. 2038 [handwritten] (NHMUK). Female: Lagos Dist. / S. Nigeria / IBD. 4.1949 / W.K.S Merrett [rinted, date partially handwritten]; Brit. Mus. / 1962-270 [printed] (NHMUK). Male: Ibadan Nig. / Dec. 59 / at light / C Phoyt [handwritten]; 3 [handwritten]; C.I.E. Coll. [printed] / A. 956 [handwritten]; Sirthenea / rapax Horv. [handwritten] / M.S.K. Ghauri det. 1966 [printed]; Pres. by / Com Inst Ent / B. M. 1966-3 [printed] (NHMUK). Female: Obudu district / Ogoja Province., / S. Nigeria. / April 1945 [handwritten]; Brit. Mus. / 1962-270 [printed] (NHMUK). **Republic of South Africa**: Male: Type / H.T. [printed on round label]; Africana / Dist. [handwritten]; Pretoria / (W.L.D.) [printed]; Distant Coll. / 1911-383 [printed]; [pinned vial with genitalia] (NHMUK). Female: C. Bon / Spei [printed]; Fry Coll. / 1905.100 [printed] (NHMUK). **Republic of Cameroon**: Female: Africa / Camerun [printed]; Sirthenea / rapax Horv [handwritten]; Holotypus; Hung. Nat. Hist. Mus. / Budapest / Coll. Hemiptera (HNHM). **Republic of Sierra Leone**: Female: S. Leona [printed on old, small label]; Sirthenea / leonina Horv. [handwritten]; Holotypus [printed]; Hung. Nat. Hist. Mus. / Budapest / coll. Hemiptera (HNHM). **Tanzania**: Female: Tang. Terr. / Ukerewe. [printed] / Father Conrads [printed]; XII [handwritten]; UR 13 [handwritten]; Sirthenea /africana Dist. [handwritten] / N.C.E. Miller det.1956 [printed, date partially handwritten] (NHMUK). Male: Zanzibar [handwritten]; Museum Paris / Coll. Noualhier 1898 [printed] (MNHN). **Uganda**: Female: UGANDA / Bukyamata, / J.J. Ruwabuneza. / B.M. 1967-677. [handwritten] (NHMUK). **Zambia**: Female: Katambo / Zambia / 1-II-1965 [handwritten] / Nat. Museum S. Rhodesia [printed]; Brit. Mus. [printed] / 1970 - 289 [partially handwritten]; Sirthenea / africana Dist. [handwritten] / det. G.M.Black 1968 [printed] (NHMUK). Male: 33 [handwritten]; N Rhodesia / 1912 / Dr. Ll. Lloyd [printed]; Pres. by / Imp. Inst. Ent. / B.M. 1935-101. [printed] (NHMUK).

1. ***Sirthenea flaviceps* (Signoret, 1860)**

**Madagascar:** Male: Madag. [handwritten] / Coll. Signoret. [printed]; flaviceps [handwritten] / det. Signoret [printed]; Sirthenea flaviceps Sign. [handwritten]; Typus / Rasahus / flaviceps Signoret, 1860 / etik. Hecher 1996 / Redv. 559/1 (NHMW). Male: Museum Paris / Madagascar / Bezanozano / Coll. Noualhier 1898 [printed]; Type [printed on red label]; Sirthenea / flaviceps Sign. / var. lugubris Horv. / type Horváth [handwritten] (MNHN). Male: Madagascar / Antongil B. / Mocquerys [printed]; flaviceps Sign. / var. adelpha Horv [handwritten]; Syntypus [printed, label with red frame] / S. flaviceps Sign. / var. adelpha Hv., 1909 / labeled Rédei, 2007 [handwritten] (HNHM). 4 Males: Madagascar / Antongil B. / Mocquerys [printed]; Syntypus [printed, label with red frame] / S. flaviceps Sign. / var. adelpha Hv., 1909 / labeled Rédei, 2007 [handwritten] (HNHM). Male: Moramango / Madagascar / 908 m [handwritten]; flaviceps Sign. / var. lugubris Horv. [handwritten]; Hung. Nat. Hist. Mus. / Budapest / coll. Hemiptera [printed] (HNHM). Male: Madagascar / Antongil B. / Mocquerys [printed]; Sirthenea / flaviceps Sign [handwritten]; Hung. Nat. Hist. Mus. / Budapest / coll. Hemiptera [printed] (HNHM). Female: Madagascar / Antongil B. / Mocquerys [printed]; flaviceps / Sign [handwritten]; Hung. Nat. Hist. Mus. / Budapest / coll. Hemiptera [printed] (HNHM). Male: Museum Paris [printed] / Madagascar S-O. / Manantenina / Génot 1901 [handwritten] (MNHN). Male: Tongobory / Sept-Lacs / III-56 / AR. [printed]; Institut / Scientifique / Madagascar [printed] (MNHN). Female: madag [printed]; Museum Paris / Coll. G. Fallou 1895 [printed] (MNHN). Male: Station Agric / Bas-Mangoky [printed]; Institut / Scientifique / Madagascar [printed] (MNHN). Male: Madagascar Est / dct Sambava / Marojejy / Ambinanitelo 500m / XII-58 Raharizonina[printed]; Institut / Scientifique / Madagascar [printed] (MNHN). Male: Museum Paris / Madagascar / Tananarive / Waterlot 1914 [printed] (MNHN). Male: Madagascar Est / district Sambava / Marojejy / Ambinanitelo 500 m / XII-58 Raharizonina [printed]; Institut / Scientifique / Madagascar [printed on blue label]; Museum Paris / Coll Generale [printed] (MNHN). Male: Madagascar-Est / dct. Sambava / R.N.XII-Marojejy / Ambatosoratra 1000m / VII-60 P. Soga [printed]; Institut / Scientifique / Madagascar [printed on blue label]; Museum Paris / Coll Generale [printed] (MNHN). Female: Madagascar-Est / Ivontaka 8m / dctMaroan setra / III.58 Soga Raharizonina [printed]; Institut / Scientifique / Madagascar [printed on blue label]; Museum Paris / Coll Generale [printed] (MNHN). 2 Males: Manomhana / S/P.Soanieiana-Ivongo / 9/67 / Tamatave [handwritten]; ORSOM Paris [printed] (MNHN). Male: Madagascar-Est / Dist. Mananara-N / Mont Antampone / Vadon-Peyrieras [printed] / VII-1985 [handwritten]; Museum Paris / Coll Generale [printed] (MNHN). Male: Madagascar Centre / route d’Ambositra à Ambohimanga du Sud / km 39 1350m / 6/11 - XI - 1963 / P. Viette [printed]; Museum Paris [printed]; Museum Paris / Coll Generale [printed] (MNHN). Female: Bekily / Madagascar [handwritten]; Seirig. / 3-40 [handwritten]; Museum Paris / Coll Generale [printed] (MNHN). Male: Ambohitantely / Tampoketsy 1600m / Ankazobe / 27-XII-56 P. Griv. [printed]; Institut / Scientifique / Madagascar [printed on blue label]; Museum Paris / Coll Generale [printed] (MNHN). Female: Madagscar / N.W. / Amboromalandy [handwritten]; D. Wintrebert. / 1. IV. 1967 [handwritten]; Museum Paris / Coll Generale [printed] (MNHN). Male: Madagscar / N.W. / Amboromalandy [handwritten]; D. Wintrebert. / 27. IV. 1967 [handwritten]; Museum Paris / Coll Generale [printed] (MNHN). Male: Madagascar / Lambomakandro / Tuléar [printed]; Muséum Paris / 1935 / R. Catala [printed on blue label]; Museum Paris / Coll Generale [printed] (MNHN). 2 Females: Madagascar-Est / Fampanambo 25m / dst Maroanosetra / III.58 Saga-Raharizonina [printed]; Institut / Scientifique / Madagascar [printed on blue label]; Museum Paris / Coll Generale [printed] (MNHN). Male: Madagascar-Est / district Sambava / Marojejy / Ambinanitelo 500m / XII-58 Raharizonina [printed]; Institut / Scientifique / Madagascar [printed on blue label]; Museum Paris / Coll Generale [printed] (MNHN). Male: Madagascar-Est / district Sambava / R.N.XII- Marojejy / Ambatosoratra 1000m / VIII-60 P. Soga [printed]; Institut / Scientifique / Madagascar [printed on blue label]; Museum Paris / Coll Generale [printed] (MNHN). Female: Ampijaroa 120m / Ankarafantsika / 30-VIII-56 P. Griv [printed]; Institut / Scientifique / Madagascar [printed on blue label]; Museum Paris / Coll Generale [printed] (MNHN). Male: Madagascar-est / Dist. Monanara-N / Mont Antampone / Vadon-Peyriras [printed] / VIII. 1965 [handwritten]; Museum Paris / Coll Generale [printed] (MNHN). Male: Amrijoroa / Tsaramandroso [printed]; Institut / Scientifique / Madagascar [printed on blue label]; Museum Paris / Coll Generale [printed] (MNHN). Male: Madagascar-Est / Ambodivoangy 20m / dct Maroantsetra / III.53 Soga-raharizonina [printed]; Institut / Scientifique / Madagascar [printed on blue label]; Sirthenea / flaviceps / Sign. [handwritten] / A. Villiers det 195 [printed]; Museum Paris / Coll Generale [printed] (MNHN). Female: Madagascar Nord-Ouest / dct. Majunga / forêt Ankarafantsika 120m / XII-59 / Raharizonina [printed]; Institut / Scientifique / Madagascar [printed on blue label]; Museum Paris / Coll Generale [printed] (MNHN). Male: périnet [printed on]; Institut / Scientifique / Madagascar [printed on blue label]; Museum Paris / Coll Generale [printed] (MNHN). Male: Fort-Dauphin; Viany B. / II-56 A. [printed]; Institut / Scientifique / Madagascar [printed on blue label]; Museum Paris / Coll Generale [printed] (MNHN). Male: Madagascar-Est / Fampanambo 25m / dct Maroantsetra / III.58 Soga-raharizonina [printed]**;** Institut / Scientifique / Madagascar [printed on blue label] (MNHN). Male: Sakaraha / Lambomakandro / P. Griveaud [printed]; Institut / Scientifique / Madagascar [printed on blue label] (MNHN). Female: Maroantsetra / Ambodivoangy / J.V. [printed]; Institut / Scientifique / Madagascar [printed on blue label] (MNHN). Female: Madagascar / Reg Maroantsetra / 1.56 Vadon! [printed, date handwritten] (MNHN). Male: Museum Paris / Madagascar / E. Dorr 64-97 [printed] (MNHN). Female: Namoroka / IX.52 (R.P.) [handwritten]; Institut / Scientifique / Madagascar [printed on blue label] (MNHN). Male: Madagascar / Vohemar / Coll. Le Moult [printed]; Coll. V [handwritten]; Collection / E. de Bergevin [printed]; 4800 [handwritten]; Sirthenea flaviceps Sign / v. adelpha Horv / Madagascar [Handwritten] (MNHN). Male: Museum Paris / Madagscar / Mandraka / Entre Tananarive et Tamatave / Forêt Manjakandriana / A. Mathiaux 1910 [printed] (MNHN). Female: Madagascar / Mananjary [handwritten]; Museum Paris [printed] / R. Catala [handwritten, blue label] / Museum Paris / Coll Generale [printed] (MNHN). Male: Madagascar Centre / route d’Ambositra à Ambohimanga du Sud / km 39 1350m / 6/11 - XI - 1963 / P. Viette [printed]; Museum Paris [printed]; Museum Paris / Coll Generale [printed] (MNHN). 2 Males: Bekily / Madagascar [handwritten]; Seirig. / 3-40 [handwritten]; Museum Paris / Coll Generale [printed] (MNHN). Female: Bekily / Madagascar [handwritten]; Seirig. / 3-40 [handwritten]; Museum Paris / Coll Generale [printed] (MNHN). Female: Madaga [printed on blue, round]. Museum Paris / Coll. G. Fallou 1895 [printed on blue]; Museum Paris / Coll Generale [printed] (MNHN). Male: Madagascar / Forét Cote Est [printed]; Muséum Paris / 1934 / R. Catala [printedon blue label]; label with male genitalia; Museum Paris / Coll Generale [printed] (MNHN). Male: Antananarivo / Madag. Sikora [handwritten on old, blue label]; Sirthenea ♂ / flaviceps Sign. / f. lugubris Horv.[handwritten] / det. Hedicke 1947 [printed, date partially handwritten] (ZMHB). Female: flaviceps / var. Sign. [handwritten]; Antan / Madagas [handwritten]; Distant Coll. / 1911-383 [printed]; Sirthenea / flaviceps Sign. / var. lugubris / Horv. [handwritten] / det. R.J.Izzard 1937 [printed, date partially handwritten] (NHMUK). Male: SW Madagascar / Isalo N. P., Zahavola for. / cca 850 m; 21.i.2013 / M. Trýzna leg. [printed] (MMBC). 2 Males: Madagascar 2011 / Ambohitantely Spec. Res. / S18º11’51”E17º17’03” / 1530m; at light, 24–29.xi. / P. Baňař lgt. [printed] (MMBC).

1. ***Sirthenea* *picescens* Reuter, 1887**

**Madagascar**: Female: Madagascar / Nossi-Bè [printed on]; Coll. / Brancsik [printed on]; Sirthenea (Mono- / gmus) picescens / REUTER, 1887 [handwritten] / det Rédei D., 2007 [printed, recent label]; Hung. Nat. Hist. Mus. / Budapest / coll. Hemiptera [printed on recent label] (HNHM). Male: Madagascar / Antongil B. / Mocquerys [printed]; Syntypus [printed]/ Sirthenea atro- / cyanea Horv., 1911 / labelled Rédei, 2007. [handwritten, recent label]; Hung. Nat. Hist. Mus. / Budapest / coll. Hemiptera (HNHM). Male: Madagascar / Antongil B. / Mocquerys [printed]; Sirthenea / atrocyanea Horv. [handwritten]; Syntypus [printed]/ Sirthenea atro- / cyanea Hv., 1911 / labelled Rédei, 2007. [handwritten, recent label]; Hung. Nat. Hist. Mus. / Budapest / coll. Hemiptera (HNHM). Male: Madagascar / Antongil B. / Mocquerys [printed]; Syntypus [printed]/ Sirthenea atro- / cyanea Hv., 1911 / labeled Rédei, 2007. [handwritten, recent label]; Hung. Nat. Hist. Mus. / Budapest / coll. Hemiptera (HNHM). Female: Mada- / gascar. [printed]; Sikora. [printed] (NHRS). Male: Madagascar-Est / Dist. Mananara-N / Serenambe / Vadon-Peyrieras [printed] / VIII. 1965 [handwritten]; Museum Paris / Coll. Generale [printed] (MNHN). Female: Environs de / Rogez / Madagascar; Museum Paris / Coll. Generale (MNHN). Male: Madagascar Est / Perinet / A. Peyrieras X-1972 [printed]; Museum Paris [printed] (MNHN). Female: Madagascar Est / Perinet / A. Peyrieras X-1972 [printed]; Museum Paris [printed] (MNHN).

1. ***Sirthenea* *rodhaini* Schouteden, 1913**

**Democratic Republic of Congo**: Male: Holotypus; Musée du Congo / Sokele [printed] / 31-XII-1911 [partially handwritten] / Dr. Bequaert [printed]; R. Dét [printed] / D [handwritten] / 2453 [printed]; Sirthenea / Rodhaini / Sch. / Type [handwritten] (RMCA). Male: Holotypus [printed]; Museé du Congo / Mayombe [printed] :Tshela [handwritten] / 6-V-1924 [partially handwritten] / A. Collart [printed]; R. Dét. [printed] / G [handwritten] / 2453 [printed]; Sirthenea / collarti Sch. / Type [handwritten] (RMCA). Male: Coll. Mus. Congo / Tshuapa : Ikela / 1956 / R. P. Lootens [printed] (RMCA). Brachypterous Female: Coll. Mus. Congo / Mayidi / 1945 / Rév.P.Van Eyen [printed]; Sirthenea / collarti Sch. [handwritten] (RMCA). Brachypterous Female: Coll. Mus. Congo / Mayidi / 1942 / Rév.P.Van Eyen [printed]; Sirthenea / collarti Scht / Det. Schouteden [handwritten] (RMCA). Female: Biot. No 64 / Ilôt de forêt / marécag. Inondée [printed]; I.R.S.A.C.–Mus. Congo / Kwango: terr. De Feshi, / rive g. Kwenge II-1959 / B. 64 Mme J. Leleup [printed] (RMCA). **Republic of Angola**: Female: Ang. 10625; Marco de Canavezes / Angola III-56; Type; *Sirthenea / angolana* / A. Villiers det. 1957 (MNHN). Female: Angola (A26) / Salazar, I.I.A.A. / 9–15.iii.1972; at light; Southern / African Exp. / B.M. 1972-I (NHMUK). Male: Angola (A30) / 7 mls. W. Gabela / 16–18.iii.1972; at light; Southern / African Exp. / B M. 1972-I (NHMUK). **Republic of Cameroon**: Male: Abong-Mbang [printed] / 2-V-68 [handwritten]; Muséum Paris / Cameroun / B. de Miré [printed]; Sirthenea / collarti / Schout [handwritten] / A Villiers det 1968 [partially hanwritten] (MNHN). Male: N Koemuone / 24-VI-66 [handwritten]; Muséum Paris / Cameroun / B. de Miré [printed] (MNHN).

1. ***Sirthenea* *caiana* Chłond, 2008**

Male: N Vietnam 1986 / prov. Vinh phu / Tam dao 27.5.–2.6. / V Švihla lgt; Collectio / National Museum / Praha, Czech Republic; Holotype [printed on red label]; Sirthenea / caiana n. sp. [handwritten] / det D. Chłond 2008 [printed]. (NMPC). 3 Males: N Vietnam, 21,35N 106,30E / 52 km SW of Lang Son, / 27.iv.–6.v.1996,370m / Pacholátko & Dembický leg. [printed]; Sammlung–Collection / Ernst Heiss / Innsbruck – Austria (TLMF).

1. ***Sirthenea* *dimidiata* Horváth, 1911**

**Taiwan**: Male: Formosa / Sauter [printed]; Kanshirei / 908 [printed]; Sirthenea / dimidiata Horv. [printed] (HNHM). Male: Formosa / Sauter [printed]; Kanshirei / 908 [printed] (HNHM). Male: Formosa / Sauter [printed]; Mt. Hoozan [printed] / 1910. r. [partially handwritten] (HNHM). 2 Males: Formosa. / P.A. Holst / 1902-336. [printed]; dimidiata [handwritten by pencil on white label]. Male: Formosa / Sauter [printed]; Kosempo / 903. VI [printed, partially handwritten] (HNHM). **China**: 2 Males: 4400 ft. [handwritten] / ShinKaisi / Mt Omei [printed]; Szechuen / China / DC Graham (USNM). Male: 4400 ft. / ShinKaisi / Mt Omei [printed]; near Kiating / Szechuen China / DC Graham 1921 (USNM). 2 Males: Kuatun (2300 m), 40n. Br. / 117. 40ö. L. J. Klapperich / 6.6. 1938 (Fukien) [printed]; Collectio / National Museum / Praha, Czech Republic [printed] (NMPC). Male: Kuatun (2300 m), 40n. Br. / 117. 40ö. L. J. Klapperich / 22.5. 1938 (Fukien) [printed]; Collectio / National Museum / Praha, Czech Republic [printed] (NMPC). Male: Kuatun (2300 m), 40n. Br. / 117. 40ö. L. J. Klapperich / 21.6. 1938 (Fukien) [printed]; Collectio / National Museum / Praha, Czech Republic [printed] (NMPC). Male: Kuatun (2300 m), 40n. Br. / 117. 40ö. L. J. Klapperich / 5.6. 1938 (Fukien) [printed]; Collectio / National Museum / Praha, Czech Republic [printed] (NMPC). Male: Kuatun (2300 m), 40n. Br. / 117. 40ö. L. J. Klapperich / 4.6. 1938 (Fukien) [printed]; Collectio / National Museum / Praha, Czech Republic [printed] (NMPC). Brachypterous Female: Kuatun (2300 m), 40n. Br. / 117. 40ö. L. J. Klapperich / 6.6. 1938 (Fukien) [printed]; Collectio / National Museum / Praha, Czech Republic [printed] (NMPC). Brachypterous Female: Kuatun Fukien / China, 15.6.46 / leg. Tschung-Sen [printed]; Collectio / National Museum / Praha, Czech Republic [printed] (NMPC).

1. ***Sirthenea* *flavipes* (Stål, 1855)**

**Afghanistan:** Male: SW Afganistan / 35 km ndl, Kandahar / Arghandab Dam / 1150 m 23–27.V. / 1961, leg. G. Ebert [printed]; Staalsslg. / München [printed]; Collectio / National Museum / Praha, Czech Republic [printed] (NMPC). Male: SW Afganistan / 35 km ndl, Kandahar / Arghandab Dam / 1150 m 23–27.V. / 1961, leg. G. Ebert [printed]; Staalsslg. / München [printed]; Collectio / National Museum / Praha, Czech Republic [printed]; Sirthenea / flavipes Stal [handwritten] / det. L. Hoberlandt, 1954 [printed, date partially handwritten] (NMPC). Male: J. Klapperich / Kandahar-Kuna / 950 m, 24.1.53 / S-Afganistan [printed]; Collectio / National Museum / Praha, Czech Republic [printed]; Sirthenea / flavipes Stal [handwritten] / det. L. Hoberlandt, 1964 [printed, date partially handwritten] (NMPC). Male: 9–11.7.1965 / NO. Afganistan / Petso-Tal O v. / Schari-Sarai, 1100m / Kasy & Vartian [printed]; Ectomocoris / spec. ? [handwritten]; E. Wagner det. 1988 [printed] (NHMUK). **Bangladesh**: 2 Males: Bangladeš 5.73 / Khulna env. / J. Krystl lgt [printed]; Ex Collectio / Z. Jindra, Prague [printed] (ZJPC). **Cambodia:** Female: Cambodia / Pailin 200m a.s.l./ 11.V.2009 / S. Murzin lgt. [printed]; Collectio / National Museum / Praha, Czech Republic [printed] (NMPC). **China**: Male: Kiukiang / China14.3. unreadable text / Hafang unreadable text [handwritten]; Otto / Knauss [handwritten]; Sirthenea / flavipes Stal [handwritten]; det. W. Stichel jr. [printed] (ZMHB). Female: Shang / hai [handwritten]; 27/6 / 05 [handwritten]; Mus. / Hauschild. / 12-9-1914 [printed] (NHMD). Male: Shang / hai [Handwritten]; Mus. / Hauschild. / 12-9-1914 (NHMD). Male: B. / Piaroso [handwritten]; Mus. / Hauschild. / 12-9-1914 (NHMD). Female: Pingshiang / Süd-China / Dr. Kreyenberg [printed]; Kiritshenko det. [printed] (SMF). Male: Pingshiang / Süd-China / Dr. Kreyenberg [printed]; Sirthenea / Flavipes Stål [handwritten] / kiritshenko det. [printed] (SMF). Female: Shanghai / China [printed]; EDeschamps / collector [printed] (USNM). Female: China, Guangdong prov. / Danxia Shan NP, 23.iv.2013 / (garden at light) / 25º02.4’N, 113º45.0’E, 100 m / J. Hájek & J. Růžička leg. [printed]; Collectio / National Museum / Praha, Czech Republic [printed]; Sirthenea / flavipes (Stål, 1855) [handwritten]; det. Rédei D., 2014 [printed] (NMPC). Female: China, Jiangxi prov., 26.iv.2011 / Jinggang Shan Mts / Xiangzhou viii. Env. / (rice fields; forested stream valley) / 26º35,5’N, 114º16,0’E, 374 m / M Fikáček & J. Hájek leg. [printed]; Collectio / National Museum / Praha, Czech Republic [printed] (NMPC). Female: China / Yunnan [printed] (HMNH). Female: Annam / Phuc-Son / Nov. Dez. / H. Fruhstorfer [printed]; flavipes Fabr. / var. apicalis Sign. [handwritten] (HMNH). Female: Kinfushan . Prov. Szechuen / West-China IV/V 29. / Coll. H. Becker [printed]; Coll. Lindberg [printed]; empty yellow label; HTTP://ID.LUOMUS.FI/ / GV 44234 / China Sichuan Jinfo Shan / 28.97 N, 107.16E / 1.IV.–31.V.1929 / Becker, H. leg. [printed] (MZH). Male: Beijing / China / 1978-VI-14 [handwritten]; Sirthenea [printed] koreana [handwritten] / Lee et Kerzhner [handwritten] / Det. W. Cai. 1997-V [printed, date handwritten]; B.M.1997- [printed] / 170 [handwritten] (NHMUK). **India**: Female: Museum Paris / Inde Merid. / Trichinopoli / Coll. Noualhier 1898 [printed] (MNHN). Male: Darugiri, Garo / Hills 450 m, 19.5. [printed]; Meghalaya 1976 / Wittmer, Baroni U [printed] (NHMB). Female: India bor. or. / 19–21.V.1996 / Meghalaya [handwritten]; West Garo Hills / Bagmara env. / Jendek et Sansa lgt [handwritten]; Ex Collectio / Z. Jindra, Prague [printed]; Sirthenea [handwritten] / det. Rédei D., 2014 [printed] (ZJPC). Female: India occ., Maharashtra state / 7–11 October 2005 / Muishi env., 40 km W of Pune / F. Kantner lgt. [printed]; Ex Collectio / Z. Jindra, Prague [printed] (ZJPC). Female: Andaman Is., Havelock I. / env. of village No. 7 / 11º59N, 92º58’E / 22.iv.–14.v.1998 / Karel & Simon Majer leg. [printed]; Ex Collectio / Z. Jindra, Prague [printed] (ZJPC). Male: India 1970 / Ranchi, Bihar St. / Dr. Soběslavský [printed]; Collectio / J.L. Stehlik / Mor. Museum, Brno [printed] (MMBC). Male: India. [printed] Hemi. / Jabalpur / D. III. 83 [handwritten]; light trap [handwritten] / C.T.E.A. [printed] 15062 [handwritten] (NHMUK). Female: Hem. 1 / D. Mar. 83 / Jabalpur [handwritten]; India [printed] / light trap / CIEA 15062 [handwritten]; Sirtenea / sp. [handwritten] / det. M.S.K. Ghauri, 1983 [printed] (NHMUK). Male: NE-India: Meghalaya / Jaintia Hills reg. Jowai / 6–8.VI. 1996, 1250–1450 m / GPS N25º27’ E92º12’ (WGS84) / leg E. Jendek & O. Sausa (NHMW). Male: NE-India: Meghalaya State / W Garo Hills, Balphakram NP / 22–27.V.1996, 250–550m / GPS N25º11’ E90º51’ (WGS84) / leg. E. Jendek & O. Sausa [printed] (NHMW). Female: India, 28.VI–1.VII.1995 / Umloi SW Shillong / Meghalaya / Werner lgt. [printed]; Sammlung–Collection / Ernst Heiss / Innsbruck – Austria [printed] (TLMF). Male: India, 28.VI–1.VII.1995 / Umloi SW Shillong / Meghalaya / Werner lgt. [printed]; Sammlung–Collection / Ernst Heiss / Innsbruck – Austria [printed] (TLMF). **Indonesia:** Female: KT12/982 [handwritten on the label with specimen]; Sumatra / 12/982 / Klein [handwritten] (NHMD). Female: J. Skoygaard / Java [printed]; Java / Wedi-KL / 1902-03 [handwritten] (NHMD). Female: J. Skoygaard / Java [printed]; Java / Vedi Kltr / 14/10/1902 [handwritten] (NHMD). Male: Java [handwritten]; Sirthenea / apicalis / Signoret. / Java. [hanswritten]; Sirthenea / flavipes Stål / var. apicalis Sign. / det. Horváth [handwritten]; Museum Paris / Coll Generale [printed] (MNHN). 3 Males: Museum Paris / Java / Coll. Noualhier 1898 [pinted]. Male: Museum Paris / Java / Coll. Noualhier 1898 [pinted] (MNHN). Male: Jalajala [handwritten on old, rounded label]; Museum Paris / Java / Coll. Noualhier 1898 [pinted]. (MNHN) Female: Coll. Ciesse de Béarn / Croisiere du “Nirvana” / Java / Palaboehan Ratoe / E. Cordier 12.V. 1908 [printed, date partially handwritten]; Museum Paris / Ciesse de Béarn 1909 [peinted]; Museum Paris / Coll Generale [printed] (MNHN). Female: Coll. Ciesse de Béarn / Croisiere du “Nirvana” / Java / Soekaboemie / E. Cordier 12.V. 1908 [printed, date partially handwritten]; Museum Paris / Ciesse de Béarn 1909 [peinted] (MNHN). Female: Batavia [handwritten on old, brown, round label] / Museum Paris / Coll. G. Fallou 259-95 [printed]; Museum Paris / Coll Generale [printed] (MNHN). Female: Palabuan [ handwritten]; Museum Paris / Java / Coll. Noualhier 1898 [printed]; Museum Paris / Coll Generale [printed] (MNHN) Female: Java / Palabuan [handwritten]; Museum Paris / Coll. Noualhier 1898 [printed]; Museum Paris / Coll Generale [printed] (MNHN). Female: 3249 [printed] / Peirates glabratus N. / Java [handwritten] (ZMHB). Female: Binkam Rotts [handwritten on old labe] (ZMHB). Female: Ceylon / Hatau [handwritten on yellow label] (ZMHB). Female: Pakantan [handwritten] / Grubauer S.V. [printed, yellow label]; Pakantan [handwritten] (ZMHB). 3 Males: Indonesia: E. Sumatra / Jami Prov., Indra Hilir / env. 0–10 m / 2.2006, leg. St. Jakl [printed]; Sirthenea / flavipes (Stål) / v. apicalis Horváth [handwritten] / det. D. Chlond 2010 [printed, date partially handwritten] (NHMW). Female: Indonesia: E. Sumatra / Jami Prov., Indra Hilir / env. 0–10 m / 2.2006, leg. St. Jakl [printed]; Sirthenea / flavipes (Stål) / v. apicalis Horváth [handwritten] / det. D. Chlond 2010 [printed, date partially handwritten] (NHMW). Male: Indonesia: E. Sumatra / Indra Giri Hilir env. / coastal areas. 0–50 m / 3.2006, leg. St. Jakl [printed]; Sirthenea / flavipes (Stål) / v. apicalis Horváth [handwritten] / det. D. Chlond 2010 [printed, date partially handwritten] (NHMW). Female: Bali, Bedugul / Reg. Tamblingan Lakes / N.R., 1300m / 5.2005, leg. St. Jakl [printed]; Sirthenea / flavipes (Stål) / v. apicalis Horváth [handwritten] / det. D. Chlond 2010 [printed, date partially handwritten] (NHMW). Male: Bali, Bedugul / Reg. Tamblingan Lakes / N.R., 1300m / 5.2005, leg. St. Jakl [printed]; Sirthenea / flavipes (Stål) / v. apicalis Horváth [handwritten] / det. D. Chlond 2010 [printed, date partially handwritten] (NHMW). 3 Males: Key Tual / Rohde / ex coll.H.Fruhstorfer [printed]; Sirthenea / flavipes (Stål) / v. apicalis Horváth [handwritten] / det. D. Chlond 2010 [printed, date partially handwritten] (NHMW). Female: Dleiffer / Java [hsndwritten]; Coll. Nat. Mus. Wien [printed on yellow label]; Sirthenea / flavipes (Stäl) [handwritten] / Det L.Willemse 1982 [printed, date partially handwritten]; Sirthenea / flavipes (Stål) / v. apicalis Horváth [handwritten] / det. D. Chlond 2010 [printed, date partially handwritten] (NHMW). Female: Baron / Warsberg / Java / 1868 [handwritten]; Sirthenea / flavipes (Stål) / v. apicalis Horváth [handwritten] / det. D. Chlond 2010 [printed, date partially handwritten] (NHMW). Male: W-Sumatra 30.8. / Siberut Isl. 1992 / Saliguma [printed]; Indonesia 1992 / leg. Barries&Cate [printed]; Sirthenea / flavipes (Stål) / v. apicalis Horváth [handwritten] / det. D. Chlond 2010 [printed, date partially handwritten] (NHMW). Female: So. O. Borneo [handwritten]; Sirthenea / flavipes / Stål [handwritten]; Coll. / Breddin [printed] (Frankfurt). Male: S.O. Borneo [handwritten]; Coll. / Breddin [printed] (SMF). Female: Java [printed]; Coll. Breddin [printed] (SMF). Female: Museum Paris / Borneo / Lohaban / R. Oberthur 1898 [printed]; Museum Paris / Coll Generale [printed] (MNHN). Male: 9729 [printed]; Sampit / Borneo Rupert [printed on yellow label] (ZMHB). Female: Borneo / M. Achmidt [printed on yellow label] (ZMHB). Male: SO Borneo / Walmes S / Wolf v. Schönberg V. [printed on yellow label] (ZMHB). Female: F. Baczes / 1886 / Borneo [printed]; Sirthenea / flavipes (Stål) / v. apicalis Horváth [handwritten] / det. D. Chlond 2010 [printed, date partially handwritten] (NHMW). Male: Long / Navang [printed]; O. Borneo / Mjöberg [printed]; Swedish Museum / of Natural history / Stockholm / NHRS [printed] (NHRS). Female: Kota / Tjane [printed]; Sumatra / Mjöberg [printed]; Swedish Museum / of Natural history / Stockholm / NHRS [printed] (NHRS). 3 Males: Indonesia / Java, Kruvang [printed]; III 1955 / EDresner [printed] (USNM). Male: Indonesia, E Kalimantan / ca. 55 km W of Balikpapan / PT Fajar Surya Swadaya [area] / 01º16.4’S, 116º21.1’E, 82 m / J. Hájek, J. Schneider & / P. Votruba leg. 23.xi.–2.xii.2011 [printed]; base camp surrounded with / Acacia plantation: individual / collecting on vegetation, dead / wood and in puddles, + light trap [printed]; Collectio / National Museum / Praha, Czech Republic [printed] (NMPC). Male: Indonesia, April–May 2005 / Mentawai Isls., Siberut Isl. / Salappa env. 50–100 m / St. Jaki lgt [printed]; Ex Collectio / Z. Jindra, Prague [printed] (ZJPC). Male: East Sumatra, 0–50 m / coastal area of / Indra Giri Hilir env. / 3.2006, St. Jaki lgt. [printed]; Ex Collectio / Z. Jindra, Prague [printed] (ZJPC). Male: Indonesia Lesser Sundas / 9–15 November 2006 / Lomblen Isl., Lamaiera vill. env., S. Jaki lgt. 100–500 m [printed]; Ex Collectio / Z. Jindra, Prague [printed] (ZJPC). 2 Males: Indonesia / Flores, 1200m / Ruteng / 27-XI-1961 / J.M.A. Groenendael [printed] (RMNH). 2 Females: Key Tual / Ronde / ex coll H. Fruhstorfer [printed]; Coll / Breddin [printed] (SMF). Male: O. Borneo / Mjöberg [printed]; Swedish Museum / of Natural History / Stockholm / NHRS [printed] (NHRS). Male: Key Tual / Rohde / ex. Coll. H. Fruhstorfer [printed] (NHMW). Male: W-Sumatra 30.8 / Siberut Isl. 1992 / Saliguma [printed]; Indonesia 1992 / leg. Barries & Care [printed] (NHMW). 3 Females: Sumatra [printed]; Coll. Brancsik [printed on ble label] (HMNH). Male: Sumatra [printed]; Coll. Brancsik [printed on ble label] (HMNH). Male: W-Sumatra 30.8 / Siberut Isl. 1992 / Saliguma [printed]; Indonesia 1992 / leg. Barries & Care [printed] (NHMW). 3 Females: Sumatra [printed]; Coll. Brancsik [printed on ble label] (HMNH). Male: Sumatra [printed]; Coll. Brancsik [printed on ble label] (HMNH). Male: Java Orient. / Lawang [handwritten] (HMNH). 5 females: Borneo / Xántus [printed] (HMNH). Male: Denpasad / Insel Bali / Holl. Indien. [handwritten] (HMNH). Female: East Sumatra, March 2005 [handwritten]; Indra Giri Hilir env. / coastal area / S. Jakl lgt. [handwritten]; Ex Collectio / Z. Jindra, Prague [printed] (ZJPC). 2 Males: Indonesia, April–May 2005 / Mentawai Isls., Siberut Isl. / Salappa env. 50–100 m / St. Jaki lgt [printed]; Ex Collectio / Z. Jindra, Prague [printed] (ZJPC). Female: Indonesia, W Sumatra / 13 Febr. 1999, Mt. Singgalang / Annai Valley env., 600 m / S. Jaki lgt. [printed]; Ex Collectio / Z. Jindra, Prague [printed] (ZJPC). Female: Celebes [handwritten]; Sirthenea sp. [handwritten] / Det. N.C.E. Miller 1952 [printed; date partially handwritten]; HTTP://ID.LUOMUS.FI/ / GV 44232 / Indinesia Sulawesi [printed] (MZH). **Iran**: Female: SE Iran, 11–12.4.73 / Ghasemabad, 10 km / Bampur (vall.) [printed]; loc no 157 / Exp. Nat. Mus. / Praha [printed]; Collectio / National Museum / Praha, Czech Republic [printed] (NMPC). Female: Iran, 10–11.IV.2000 / Sistan va baluchestan Prov. / 25 km S Kahiri, 1050 m, (26º44’N, 61º04E), (light) [printed]; Iran 2000 Czech Biological / Expedition / J. Hájek & M. Mikát leg. [printed]; Collectio / National Museum / Praha, Czech Republic [printed] (NMPC). **Japan**: Male: Museum Paris / Japon / Avomori & Hirosaki / R. oberthur 1898 [printed]; Museum Paris / Coll Generale [printed] (MNHN). Male: Myadzu / Japon [handwritten on yellow label]; 4735 [handwritten]; Collection E. de Bergevin [printed] (MNHN). Male: 9933 [printed]; Japonia / Dönitz [handwritten on yellow label] (ZMHB). Male: Japan / Dönitz [printed] (ZMHB). Female: Matsuyama / Ehime Pref. / VI-28.1958 / light trap [handwritten]; Collectio / National Museum / Praha, Czech Republic [printed] (NMPC). Male: Heianza / Okinawa / July 30, 1945 / A.B. Hardcastle [handwritten] (USNM). Male: Kawayoshihara / Ishigaki / 30.V.1985 [handwritten]; Ex Collectio / Z. Jindra, Prague [printed]; Sirthenea / flavipes / (Stål, 1855) [handwritten] (ZJPC). **North Korea**: Male: Korea – Wonsan / VI. 1955 – leg. Inż. Leśn. Witold Ja- / mrógiewicz [handwritten]; Coll S. Kapuściński [printed]; Collectio / National Museum / Praha, Czech Republic [printed] (NMPC). Male: Plason [printed] / 1886 [partially handwritten] / Korea [handwritten]; Sirthenea / flavipes (Stål) [handwritten] / det. D. Chlond 2010 [printed, date partially handwritten] (NHMW). Male: Dr Sobǎslavsky / Fenjan, Korea / 1961. [printed]; Collectio / National Museum / Praha, Czech Republic [printed] (NMPC). **South Korea**: Male: Rep. Korea / Suweon / near Seoul / 7.vi. 1974 / P.E.S. Whalley / B.M. 1974-392 [printed, date partially handwritten]; Rep. Korea / Suweon / near Seoul / July 1974 / P.E.S. Whalley / B.M. 1974-392 [printed, date partially handwritten] / at light [handwritten] (NHMUK). **Laos**: Male: Laos north, 13–24.V.1997 / 16 km NW Louang Namtha / N 21º07.6,E101º21,0 / alt.750±100 m; E.Jendek & O. Šauša leg. [printed]; Sirthenea / flavipes (Stål) / v. apicalis Horváth [handwritten] / det. D. Chlond 2010 [printed, date partially handwritten] (NHMW). Male: Laos: N-Vientiane Prov. / Vang-Vieng 300 m, N 18º55’23”, E 102º26’55” / 10–15.v.& 01–06.vii.2001 / Jiři Kolibáč leg. [printed]; Entomological expedition / “Laos 2001" / Moravian Museum Brno / Czech Republic [printed] (MMBC). Female: Laos, Boli Kham xai prov. / 18º16’N 103º11’E / 70 km NEE of Vientiane, 27–30.iv.1997 150 m, / Vit Kubáň leg. [printed]; Vit Kubáň expedition / “Laos 1997” / Moravian Museum Brno / Czech republic [prined] (MMBC). Female: Laos, Vientianne prov., / Lao Pako env., 200 m, 55 km NE of Vientiane / 19–22.v.2004, J. Bezděk leg. [printed]; Coll. Petr Baňař / [petrbanar@seznam.cz](mailto:petrbanar@seznam.cz) [printed] (MMBC). Female: Laos-N, 21.iv.1999 / Louang, Phrabang prov., / 19.53’N 102.09’E, / Khan riv., 300m / Vit Kubáň leg. [printed]; Vit Kubáň expedition / “Laos 1999” / Moravian Museum Brno / Czech republic [prined] (MMBC). Male: Vientiane (Laos) [printed]; Museum Paris / Collection / Dispons [printed] (MNHN). Female: Lanlou / china [handwritten]; Ross commut. [handwritten]; Subfam / Peiratinae / ? spec [handwritten]; Kiritshenko det [printed] (SMF). Female: Laos / Muong Om / 19.I.1919 / R.V. de Salvaza [printed] (NHMUK). Female: Laos centr. Khammouan prov. / 4–16.XI., 25–30.XI.2000 / Ban Khoun Ngeun env. / N 18º07’, E 104º29’, alt. 250m / E. Jendek & P. Pacholatko leg. [printed] (NHMW). **Malaysia** Male: Sarawak III. 1994 / Rumah Kabau anak muggot / Ng sebong Beleh / 25 km E. Kapit.J.Kodada [printed]; S. flavipes [handwritten on red]; Sirthenea / flavipes (Stål) / v. apicalis Horváth [handwritten] / det. D. Chlond 2010 [printed, date partially handwritten] (NHMW). Male: Malaysia: banom Mts: / 15km E.Kampang Dang: 700m / 3.53N 102,01E;1.iv.1998 /Dembicky&Pacholatko leg.[printed]; Sirthenea / flavipes (Stål) / v. apicalis Horváth [handwritten] / det. D. Chlond 2010 [printed, date partially handwritten] (NHMW). Female: Sarawak, Kapit distr. / Sebong, Baleh riv. / 6–21.3.1994 / Sv. Bily lgt. [printed]; Collectio / National Museum Praha, Czech Republic [printed] (NMPC). Male: Sarawak, Kapit dist. / Rumah Ugap vill. / Sut. Riv. 3–9.3.1994 / Sv. Bily lgt. [printed]; Collectio / National Museum / Praha, Czech Republic [printed] (NMPC). Female: Malaysia, Sarak / Sipitang, Mendolong / leg. S. Adebratt [printed]; Swedish Museum / of Natural history / Stockholm / NHRS [printed] (NHRS). Male: N. Borneo / Bettotan / Nr Sandakas [printed] / Aug 11–14 [handwritten] 1937. [printed]; Ex. F.M.S. / Museum / B.M. 1955-354 [printed] (NHMUK). Male: N. Borneo / Bettotan / Nr Sandakas [printed] / July 29 [handwritten] 1927. [printed]; Ex. F.M.S. / Museum / B.M. 1955-354 [printed] (NHMUK). Male: N. Borneo / Bettotan. / R. Sandakan. [printed] / Aug 5–11 1927 [partially handwritten]; Ex. F.M.S. / Museum / B.M. 1955-354 [printed] (NHMUK). Male: Malaysia – Pahang / 35 km SW of Kuala Rompin / Kuala Rompin State Park / 2.617ºN, 103.337ºE 500m / E. Jendek lgt. 28.ii–13.iii.2011 [printed]; Ex Collectio / Z. Jindra, Prague [printed] (ZJPC). Male: Malaysia – Borneo – Sabah / 9 May 1999 / 30 km N of Tungku / M. Snížek lgt. [printed]; Ex Collectio / Z. Jindra, Prague [printed] (ZJPC). Female: Malaysia, Sabah / Sipitang, Mendolong / Leg. S. Adebratt [printed]; 21529 [handwritten on red]; Swedish Museum / of Natural History / Stockholm / NHRS [printed] (NHRS). Male: N. Borneo / Bettotan, / Nr. Sandakan [printed] / at light July 23 [handwritten] 1927 [printed]; Ex. F.M.S. / Museum, / B.M. 1955-345 [printed] (NHMUK). Female: N. Borneo / Bettotan, / Nr. Sandakan [printed] / at light July 23 [handwritten] 1927 [printed]; Ex. F.M.S. / Museum, / B.M. 1955-345 [printed] (NHMUK). **Myanmar**: Male: U Burma / Hopin [printed] / 28–30.8.14 / Fletcher coll. [handwritten] (NHMUK). Male: Burma: Arakan State / Myoma district / Akyab [printed] lights [handwritten] / 22 Sept. 1951 [handwritten] / G.B. Vogt [printed] (USNM). Female: XI. 1996 / Burma–Rangoon / Taukkyan / klicha M., lgt [printed]; Ex Collectio / Z. Jindra, Prague [printed] (ZJPC). Female: IV. 1997 Burma / Rangoon Distr. / Hlegu–Goygon / Klicha M. lgt. [printed]; Ex Collectio / Z. Jindra, Prague [printed] (ZJPC). **Nepal**: Male: Nepal / Rapti Tal 300 m / Megouli I.IV.62 / leg. G. Ebert [printed]; Collectio / National Museum / Praha, Czech Republic [printed] (NMPC). Female: Nepal, Rapti Tal / Jhawani 200 m / V. 1967 leg. / Dieri–Forster–Schacht; Staatssig, München [printed]; Collectio / National Museum / Praha, Czech Republic [printed] (NMPC). Male: Katmandu / 24.5.–21.6.76 [printed, date partially handwritten]; Nepal, W. Wittmer / C. Baroni Urbani [printed] (NHMB). Male: C-Nepal, 21–27/7.2000 / Chitwan (Roy. Nat. Park) / Sauraha viii, 166 m / 27,35 N 84.30 E [GPS] env., / David Král lgt. (at light) [printed]; Nepal Expedition / Jan Farkač, David Král / & Jan Schneider, 2000 [pronted]; Collectio / National Museum / Praha, Czech Republic [printed] (NMPC). II instar nymph: Nepal. Prov. Bheri / Nepalganj. Rapti river / near airport, 200mNN / 30.V.1997, leg. Hartmann [printed]; collection / Naturkunde- / museum Erfurt [printed on yellow label] (NMEG). 3 III instar nymphs: Nepal, District Bheri / Nepalganj, 30.05.1997, ca / 200 mNN, Ufer Rapti- / River, Nähe Flugplatz / leg. Grill HF. [printed]; collection / Naturkunde- / museum Erfurt [printed on yellow label] (NMEG). 3 Males: Nepal, Prov. Narayani / Sauraha Rapti River / Ufer 180 mNN, 27º34’ / 80”N 84º29’49”E / LF, 18.IV.2000 / leg A. Weigel [printed]; Sirthenea / flavipes / (Stål) [printed]; collection / Naturkunde- / museum Erfurt [printed on yellow label] (NMEG). Female: Nepal: Prov. Bagmati / Kathmandu, Bagmati / nr. Gorkhana Park, / 27º43,01’N 85º18,46’E / 1340mNN, 17.VI.1999 / leg. M. Hartmann [printed]; collection / Naturkunde- / museum Erfurt [printed on yellow label] (NMEG). Male: Nepal, Prov. Nara- / Yani, Sauraha, Rapti / River, 180 mNN, F / 27º34,51”N, 84º29’30”E, 14.IV.2001, / leg. A. Weigel [printed]; collection / Naturkunde- / museum Erfurt [printed on yellow label] (NMEG). Female: Nepal, Rapti Tal / Jhavani 200 m / V. 1967 leg. / Dietl–Forster–Schacht / Staatsslg. / München [printed]; Collectio / National Museum / Praha, Czech Republic [printed] (NMPC). Male: Het / 123 [handwritten]; Nepal / Kathmandu [printed] / V. 1973 / N. Kumar [handwritten] / C.I.E. A 6892 [printed]; Sirtenea sp. [handwritten]; det. M.S.K. Ghauri, 1975 [printed] (NHMUK). Female: LL II [handwritten]; Peradeniya, / Ceylon [printed] V.II [handwritten] (NHMUK). Female: Ceylon / 18 [printed]; flavipes [handwritten] / det. Horváth [printed] (NHMW). **Pakistan**: Female: East Pakistan (KP 22) / Chittagong, Nasirabad, H.S., on light, 18–22 / leg. Fr. Dvořák I.1970 [printed]; Collectio / J.L. Stehlik / Mor. Museum, Brno [printed] (MMBC). Female: East Pakistan (KP 23) / Chittagong, Nasirabad, H.S., on light, 23–28 / leg. Fr. Dvořák I.1970 [printed]; Collectio / J.L. Stehlik / Mor. Museum, Brno [printed] (MMBC). **Philippines**: Male: Philippines, Luzon / Mt. Prov Chatol / 1800 m, 20.ix / 1988, K. Cerny lgt. [printed]; Coll. Petr Baňař / petrbanar@seznam.cz [printed] (MMBC). Female: Bay Laguna / Prov P1 [printed]; PLStangl / collector [printed] (USNM). Male: Philipines / Samar [printed] / May 1945 [partially handwritten] / J. Laffoon [printed] (USNM). Female: Nov. [printed]; Bacoor / P1 [printed]; PLStangl / collector [printed] (USNM). **Sri Lanka**: Female: MacKavoed / Ceylon 86 [handwritten]; flavipes [handwritten]; det. Horváth [printed]; Sirthenea / flavipes (Stål) [handwritten] / det. D. Chlond 2010 [printed, date partially handwritten] (NHMW). Male: Ceylon [handwritten] / Coll. Signoret [printed]; flavipes [handwritten] / det. Stal [printed]; flavipes [handwritten] / det. Horváth [printed]; Sirthenea / flavipes Stål [handwritten]; Coll. Nat.Mus. Wien [printed on yellow label]; Sirthenea / flavipes (Stäl) [handwritten] / Det. L.Willemse 1982 [printed, date partially handwritten]; Sirthenea / flavipes (Stål) [handwritten] / det. D. Chlond 2010 [printed, date partially handwritten] (NHMW). Male: Ratanpura / Ceylon / M. Löbell [handwritten]; Sirthenea / flavipes (Stål) [handwritten] / det. D. Chlond 2010 [printed, date partially handwritten] (NHMW). Male: 3250 [printed]; Ceylon Nieto. [handwritten]; Sirthenaea / flavipes ♂ / Stål [handwritten] / det.Hedicke 1947 [printed, date partially handwritten] (ZMHB). Female: blue quadrandular label; Ceylon [printed]/ Mus. Colombo [handwritten, blue label] (ZMHB). Female: Ceylon [handwritten] (HMNH). Female: Sri Lanka, central province / Inamalawa, 23.3.1997 / J. Kábelak, P. Koráková lgt [printed]; Collectio / National Museum / Praha, Czech Republic [printed] (NMPC). 2 Males: Sud Ceylon / Mai 1889 / H.Fruhstarfer [printed]; Coll. / Breddin [printed] (SMF). Female: Ceylon / Maha Illuppallama [printed] / 12.VII.60 [handwritten] / Dry Zone Res. Stn. [printed] / 17844 / 232 [handwritten]; Sirthenea / sp. [handwritten]; Sithenea / sp. [handwritten] / M.S.K. Ghauri det. [printed] (NHMUK). 2 Males: Ceylon / Maha Illuppallama [printed] / 12.7.60 Light / 232 [handwritten] / Dry Zone Res. Stn. [printed]; C.I.E Coll / No. [printed] 17197 [handwritten] (NHMUK). 2 Females: Sri Lanka, Kal Dist. / Agalawatta, Darton- / field, 5–6-III-1978 / M.D. Hubbard and / T. Wijesinhe [printed]; Sirthenea flavipes / (Stal) / Det. D.P. Ambrose 1999 [printed] (USNM). Male: Sri Lanka, Kal Dist. / Agalawatta, Darton- / field, 5–6-III-1978 / M.D. Hubbard and / T. Wijesinhe [printed]; Sirthenea flavipes / (Stal) / Det. D.P. Ambrose 1999 [printed] (USNM). Male: Sri Lanka: Gal. Dist. / Kenneliya Jungle / 11–16 January 1975 / blacklight [printed]; K.V. Krombein / P.B. Karunaratne / P. Fernando / N.V.T.A. Weragoda [printed]; Sirthenea flavipes / (Stal) / Det. D.P. Ambrose 1999 [printed] (USNM). Female: Sri Lanka: Ham Dist. / Palatupana Tank / 3 February 1975 [printed]; K.V. Krombein / P.B. Karunaratne / P. Fernando / E.G. Dabrera [printed]; Sirthenea flavipes / (Stal) / Det. D.P. Ambrose 1999 [printed] (USNM). Male: Sri Lanka: Ham Dist. / Palatupana Tank / 3 February 1975 [printed]; K.V. Krombein / P.B. Karunaratne / P. Fernando / E.G. Dabrera [printed]; Sirthenea flavipes / (Stal) / Det. D.P. Ambrose 1999 [printed] (USNM). Female: Sri Lanka: Kan. Dist. / Udawattakele, 2100 ft / black light / 4–5 October 1976 [printed]; Collected by: / G.F. Hevel / E. Dietz IV / S. Karunaratne / D.W. Balascoriya [printed]; Sirthenea flavipes / (Stal) / Det. D.P. Ambrose 1999 [printed] (USNM). Female: Sri Lanka: Rat. Dist. / Belihuloya, Belihul / Oya 23-III-1978 / M.D. Hubbard and / T. Wijesinhe [printed]; Collected at Black light [printed]; Sirthenea flavipes / (Stal) / Det. D.P. Ambrose 1999 [printed] (USNM). Female: Sri Lanka / Polonnaruwa / 6 May 1974 / Gans&Prasanna [printed]; Sirthenea flavipes / (Stal) / Det. D.P. Ambrose 1999 [printed] (USNM). Male: Sri Lanka: Gal. Dist. / Kanneliya / 22–24 May 1975 / S.I.Wood & J.L. Petty [printed]; Collected in / black light / trap [printed]; Sirthenea flavipes / (Stal) / Det. D.P. Ambrose 1999 [printed] (USNM). Female: Sri Lanka: Col. Dist. / Kalatuwawa / 12–15-VIII-1975 [printed]; Collected by: / Y.-M. Huang, / E.L. Peyton, / S. Karunaratne, / D.W. Balasooriya [printed]; Sirthenea flavipes / (Stal) / Det. D.P. Ambrose 1999 [printed] (USNM). 3 Males: Sri Lanka: Mon. Dist. / Mau Ara 10 mi. E / Udawalawa, 100 m, / 24–26-IX-1977 [printed]; Collected at / blacklight bulb [printed]; K.V. Krombein / P.B. Karunaratne / T. Wijesinhe / M. Jayaweera [printed]; Sirthenea flavipes / (Stal) / Det. D.P. Ambrose 1999 [printed] (USNM). Female: Sri Lanka: Amp. Dist. / Ekgal Aru Sanctuary / Jungle, 9–11 Mar1979 / blacklight [printed]; K.V. Krombein / T. Wijesinhe / S. Siriwardane / L. Jayawickrema [printed]; Restrictions Apply / NMNH—Sri Lanka / Agreement #6 [printed on purple label] (USNM). Female: Sri Lanka: Ham Dist. / Palatupana Tank / 3 February 1975 [printed]; K.V. Krombein / P.B. Karunaratne / P. Fernando / E.G. Dabrera [printed]; Sirthenea flavipes / (Stal) / Det. D.P. Ambrose 1999 [printed] (USNM). Female: Sri Lanka 20–22.12.2003 / Dambula env. / F. Kantner lgt. [printed]; Ex Collectio / Z. Jindra, Prague [printed] (ZJPC). 4 Females: Sri Lanka, Kotapala env. / 18–22 April 1994 / 6 17’ N, 80 33’E / R. Sauer lgt [printed]; Ex Collectio / Z. Jindra, Prague [printed] (ZJPC). 2 Females: Sri Lanka, Kotapala env. / 18–22 April 1994 / 6 17’ N, 80 33’E / R. Sauer lgt [printed]; Ex Collectio / Z. Jindra, Prague [printed] (ZJPC). Female: Ceylon / Maha Illupapallama [printed] / 12.VII.60 [handwritten] / dry Zone Res. Stn. [printed] / C.I.E. 17844 / 232 [handwritten] (NHMUK). Male: Ceylon / Maha Illupapallama [printed] / 12.VII.60 [handwritten] / dry Zone Res. Stn. [printed] / C.I.E. 17844 / 232 [handwritten] (NHMUK). Female: Ceylon / Maha Illupapallama [printed] / 12.7.60 light [handwritten] / dry Zone Res. Stn. [printed]; C.I.E. Coll. / No. 17197[handwritten] (NHMUK). **Thailand**: Female: Thailand: 240km / NW Bangkok, 25km / NW Lan-Sak [printed]; III.1989, 110m / at light / leg Thielen [printed]; Sirthenea / flavipes (Stål) [handwritten] / det. D. Chlond 2010 [printed, date partially handwritten] (NHMW). Male: NW Thailand 27.4 / 1991 Chomthong / J. Horák lgt. [printed]; Collectio / National Museum / Praha, Czech Republic [printed] (NMPC). Male: Thailand, Chumphon prov. / 14–21 March 1996 / Pha To env., 98º47’E, 9º48’N / K. Majer lgt.[printed]; Ex Collectio / Z. Jindra, Prague [printed] (ZJPC). **Vietnam**: 3 Females: Museum Paris / Tonkin / Reg. De Hoa-Binh / A. de Cooman 1928 [printed]; Museum Paris / Coll Generale [printed] (MNHN). Female: Museum Paris / Tonkin / Reg. De Hoa-Binh / A. de Cooman 1929 [printed, date partialy handwritten] (MNHN). Male: Museum Paris / Tonkin / Reg. De Hoa-Binh / A. de Cooman 1928 [printed]; Museum Paris / Coll Generale [printed] (MNHN). 3 Females: Museum Paris / Tonkin / Hoa-Binh / J. de Cooman / R. Oberthur 1919 [printed]; Museum Paris / Coll Generale [printed] (MNHN). Female: Museum Paris / Tonkin / Rég. De Hoa-Binh / A. de Cooman 1929 [printed]; Museum Paris / Coll Generale [printed] (MNHN). Male: Hanoi V. 65 / Lichtfall [handwritten]; 8 [handwritten] (ZMHB). Male: S. Vietnam, 18.–29.5.1994 / 14 km SW Bao Lac / P. Pacholatko & L. Dembicky leg. [printed]; Sirthenea / flavipes (Stål) [handwritten] / det. D. Chlond 2010 [printed, date partially handwritten] (NHMW). Male: Cochinch. [handwritten] / Coll. Signoret [printed]; spicalis [handwritten] / det. Signoret.[printed]; Sirthenea flavipes Stål var. apicalis Sign. [handwritten]; Typus / Rasahus / apicalis Signoret, 1862 / etik. Hecher 1996 / Redv. 560/4 [printed on red label]. (NHMW). Female: Museum Paris [printed] / Cochinchine / Prov. De Thulaumot / Katrach / Capus. 1909 [handwritten] (MNHN). 2 Females: Hanoi V. 65 / Lichtfall [handwritten]; 8 [handwritten] (ZMHB). Male: Hanoi V. 65 / Lichtfall [handwritten]; 8 [handwritten] (ZMHB). Male: N-Vietnam25.V.–10.VI / Sapa (Lao Cai) / 22’20’N 103’50’E / Leg. E. Jandek 1991; Sirthenea / flavipes (Stål) / v. apicalis Horváth [handwritten] / det. D. Chlond 2010 [printed, date partially handwritten] (NHMW). Female: S. Vietnam, 40 km NW An Khe / Buon Luoi, 14”10’N,108”30’E / 620–750 m, 28.3–12.4.1995 / leg. Pacholatko&Dembicky [printed]; Sirthenea / flavipes (Stål) / v. apicalis Horváth [handwritten] / det. D. Chlond 2010 [printed, date partially handwritten] (NHMW). Female: N. Vietnam 1986 / prov. Ha son Binh / Hoa Binh 5.–7.6. / V. Švichla lgt. [printed]; Collectio / National Museum / Praha, Czech Republic [printed] (NMPC). Male: Hoa Binh / Tonkin [handwritten] (MNHN). Female: Nang Tri / Tonkin [handwritten] (MNHN). Female: Chapa / Tonkin / Coll. J. Clement [printed]; Achat Boubee / 10.5.1958 [handwritten]; Museum Paris / Collection / Dispons [printed] (MNHN). Male: S. Viet Nam / Cam Ran Bay / V-30-68 [printed]; Coll by / T. Taylor [printed] (USNM). Female: South Viet Nam / Long Thanh / 1967 / Dwight Svhuh [printed] (USNM). Male: Tonkin / Montes Mauson / April–Mai 2–3000’ / H. Fruhstorfer [printed] (NHMW). Male: Phuc-Son / Nov. Dez. / H. Fruhstorfer [printed] (HMNH).

1. ***Sirthenea* *kali* Chłond, 2018**

**North–East India (West Bengal):** Female: Museum Paris / BHOUTAN / ANGLAIS / R. OBERTHÜR 1900; Holotype [printed on red label]; Sirthenea / kali n. sp. [handwritten] / det D. Chłond 2009 [printed] / vial with genitalia. (MNHN) Female: Museum Paris / BHOUTAN / MARIA BASTI / (M^ar^ DUREL) / R. OBERTHÜR 1898; Paratype [printed on red label]; Sirthenea / kali n. sp. [handwritten] / det D. Chłond 2009 [printed] (MNHN).

1. ***Sirthenea nigronitens* (Miller, 1958)**

**Indonesia (New Guinea)**: Female: Neth. Ind.–American / New Guinea Exped. / Rattan Camp/1500m / 04.ii. 1939 L.J.Toxopeus; Type; Sirtheneana / nigronitens / sub.gen.n. sp.n. (holotype) / N.C.E.Miller det. 1956. (RMNH).

1. ***Sirthenea nitida* Chłond, 2008**

**Laos:** Male: LAOS-N (Louangphrabang), / 11–21.v.2002, / 19º 35’N 101º58’E, / Thong Khan / ~750m, Vit Kubaň leg. [printed]; Entomological expedition / “Laos 2002” / Moravian Museum / Brno, Czech Republic [printed]; Holotype [printed on red label]; Sirthenea / nitida n. sp. [handwritten] / det D. Chłond 2008 [printed] (MMBC). Male: LAOS-N (Louangphrabang), / 11–21.v.2002, / 19º 35’N 101º58’E, / Thong Khan / ~750m, Vit Kubaň leg. [printed]; Entomological expedition / “Laos 2002” / Moravian Museum / Brno, Czech Republic; Paratype [printed on red label]; Sirthenea / nitida n. sp. [handwritten] / det D. Chłond 2008 [printed] (MMBC). Male: Laos-N, 24.iv.–16.v.1999, / Louang Phrabang prov., / 20º33–4’N 102º14’E / Ban Song, Cha (5km W), / ±1200, Vit Kubaň leg. [printed]; Vit Kubaň expedition / “Laos 1999” / Moravian Museum Brno / Czech Republic [printed] (MMBC). Male: Laos (NE): Hua Phan Prov. / Ban Saleui, Phou Pan (Mt.) / 15.IV.–14.V.2012, 20º12’N / 104º01’E, 1300–1900m, leg. / C. Holzschuh; Acqu. Nr. 2012-17 [printed] (NHMW). **China**: Male: China, Yunnan, Mengla / Longmen (Chinese letters). 1036 m / N21°16.255, E101°30.983 / 2009.V.8, Li&Cao / Ent. Mus. CAU, Beijing [printed] (CAU).

1. ***Sirthenea* *nigra* Cai & Tomokuni, 2004**

**Laos**: 4 Males: Lao–NE, Hua Phan prov., / ~20°12’N 104°01’E, / Phu Phan Mt., 1500– / 1900m, 17.v.–3.vi.2007, Vit. Kubáň leg. [printed]; Entomological expedition / „Laos 2007” / Moravian Museum Brno / Czech Republic [printed] (MMBC). **Malaysia**: 2 Males: Malaysia W., Pajiang / 50 km NE of Kuala Rompin / Endau Rompin Nat.P.,400m / G.Keriung.(Kg. Tebu Hitam) / 9.–30.iv.2008,P.Cechovsky lgt. [printed]; Sammlung–Collection / Ernst Heiss / Innsbruck – Austria [printed] (TLMF). **Vietnam**: Male: 4.V.95 [handwritten by pencil], 71[in the circle, handwritten by pencil] / Pha Din Pass. / 1400m L.T. [handwritten by pencil] (NSMT).

1. ***Sirthenea setosa* Chłond, 2018**

**Malaysia (Borneo):** Male: (4) [handwritten]; unidentified host / Quoin hill, Tawau / collector G. T. Lim / Date: April 1981 [handwritten on grey label]; C.I.E. Coll. [printed] / A. 13088 [number handwritten, small yellow label]; Sirthenea sp. [handwritten] / det. M.S.K. Ghauri, 1983 [printed, part of date handwritten] (NHMUK).

1. ***Sirthenea laevicollis* Horváth, 1909**

**Australia**: Male: Australia / N.S. Wales [printed]; Sirthenea / laevicollis Horv [handwritten]; typus [printed on label with red frame] (HNHM). Male: label with glued tarsus; Australia / N.S. Wales [printed]; typus [printed on label with red frame]; vial with dissected genitalia; Type ♂ / Sirthenea / laevicollis Horváth / dissected & drawn [handwritten] / det. M.B.Malipatil 1984 [printed, date partially handwritten] (HNHM). Male: N.S. Wales [printed] (NHRS). Female: Mt.Tam-M / bourine [printed]; Queensl. / *Mjöberg* [printed]; Swedish Museum / of Natural History / Stockholm / NHRS [printed] (NHRS). Brachypterous Female: Mapletón / 8/II/12 (handwritten); L6 [handwritten]; Queensland. / H. Hacker. / B.M. 1924-455 [printed] (NHMUK). Brachypterous female: Dr. Müller / N. Holl. / 1861 [handwritten]; Coll. Nat.-Mus. Wien [printed on yellow label]; Sirthenea / spp. [handwritten]; Sirthenea / laevicollis Horváth [handwritten] / Det. L. Willemse 1982 [printed, date partially handwritten] (NHMW). Brachypterous female: Dr. Müller / N. Holl. / 1861 [handwritten]; Coll. Nat.-Mus. Wien [printed on yellow label]; Sirthenea / laevicollis Horváth [handwritten] / Det. L. Willemse 1982 [printed, date partially handwritten] (NHMW).

1. ***Sirthenea obscura* (Stål, 1866)**

**Australia:** Male: Sidney [printed]; Stevens [printed]; obscura Stål [handwritten]; Typus [printed on red label] (NHRS). Male: Type [printed on round label with green margin]; 58–124 / Australia. [printed]; 111. Pirates glaber. [printed] (NHMUK). Male: Kanangra Walls / 60 km SE Oberon / 1000 m 28.11.1989 [printed]; Australia NSW / Kanangra Nat. Park / P. Oosterbroek / & C. Hartveld [printed] (RMNH). Male: Australia N.S.W. / Cabramatta. / 15.xi.1965 / M.I.Nikitin. / B.M. 1966–97. [printed] (NHMUK).

1. ***Sirthenea amazona* Stål, 1866**

**Bolivia**: 4 males: Bolivia – Santa Cruz dept., / 80 km NW of city of St. Cruz / Yapacani env. 25.xi 2007, O Safránek leg. [printed]; Collectio / Petr Baňař / Moravian Museum Brno [printed] (MMBC). Female: Rio Seco / 300 m / walz [handwritten]; Bolivia / Sta Cruz / Feber 1962 [handwritten] (USNM).

1. ***Sirthenea atra* Willemse, 1985**

**Paraguay**: Female: Horqueta / Paraguay / 57-10, W. 23-24, N [printed]; 44 Kilm. East / Paraguay Riv. [printed] /XII. 4. 1934 [handwritten] / Alberto Schulze [printed]; 227 [handwritten]; J C Lutz / Collection / 1961 [printed] (USNM). Male: Female: Horqueta / Paraguay / 57-10, W. 23-24, N [printed]; 44 Kilm. East / Paraguay Riv. [printed] /XII. 4. 1934 [handwritten] / Alberto Schulze [printed]; 227 [handwritten]; J C Lutz / Collection / 1961 [printed] (USNM).

1. ***Sirthenea dubia* Willemse, 1985**

**Argentina**: Female: S. Tomé / Corrientes [printed] / 1925 [handwritten]; Col. / G. Pallerano [printed] (MACN). Male: E. Rios / XII 1971[handwritten]; Colección / Dr. carpintero / Argentina [printed]; paratype [printed on orange label]; vial with genitalia; Sirthenea / dubia sp. nov. [handwritten]; Det. L. Willemse, 1982 [printed] (MACN).

1. ***Sirthenea ferdinandi* Willemse, 1985**

**Argentina:** Male: Argentina – LA RIOJA; Sierra d. Velasco / Huaco Arriba – Rio Huaco / ca. 1700 m 22.-23.XI.1999 / PETR ZABRANSKY LEG [printed] (NHMW). **Bolivia**: Male: Santa Cruz-Bolivia / Prov.: Florida / Loc. Pampagrande / 9-X-2007 [printed]; Leg./ Fray A. Langer [printed] (NMEG). Male: Santa Cruz-Bolivia / Prov.: Florida / Loc. Pampagrande / 10-X-1995 [printed]; Leg./ Fray A. Langer [printed] (NMEG). Female: StaCruz / Prov: Andrez Ibañez / Loc: Jardin de laso / Delicias / 7-V-1998 [handwritten]; Leg: / Steffen Reichle / Mayra Maldonado [handwritten] (NMEG). Male: Ledesma – Jujuy / 28-I958 / Torres-Ferreyra col. [printed] (MLPA).

1. ***Sirthenea jamaicensis* Willemse, 1985**

**Jamaica**: Female: Round label; Jamaica [handwritten]; Holotype [printed on orange label]; Vial with genitalia; Sirthenea / jamaicensis sp. nov. / (holotype) ♀ [handwritten] / Det. L. WIllemse 1982 [printed] (NHMUK).

1. ***Sirthenea ocularis* Horváth, 1909**

**Bolivia**: 3 Males: Dpto: Santa Cruz / Prov. Adres Ibaniez / Loc: P. de Guenda . 26-IX-1992 / Bolivia [printed]; Coll. Paolo [printed] / Battella [handwritten] (NMEG). Male: Dpto: Santa Cruz / Prov. Ichilo / Loc Parque Amboro [printed]; 4-X-1991 [handwritten]; Col: Joje Luis / Rio Saguayo / Alt: 350 m [handwritten] (NMEG).

1. ***Sirthenea pedestris* Horváth, 1909**

**Argentina**: Male: Ladesma – Jujuy / 28.I.1958 / Torres-Ferreyra col. [printed] (MLPA). **Brasil**: 2 Males: Brasil / Rio Grande do Sul / Guarani [?] / XII 1940 / leg. J. Pitoń [printed]; 5977/2521 / coll. Upper Silesian Museum / (USMB) Bytom, Poland [printed] (USMB).

1. ***Sirthenea peruviana* Drake & Harris, 1945**

**Ecquador**: Male: Ecquador, Napo /Prov. Limoncocha / 8 June 1977 / Dave L. Vinvent [printed] (USNM). Male: Ecuador Past. / Puyo, 15 May 1977 / Blacklight / P.J. Spangler & D.R. Givens #47 [printed] (USNM).

1. ***Sirthenea plagiata* Horváth, 1909**

**Ecquador**: 4 Males: Ecuador, Past. / Puyo, 11 May 1977 / Blacklight / P.J. Spangler & D.R. Givens #36 [printed] (USNM). Female: Ecuador 22-27. 11. 2004 / prov. Pastaza Santa Clara / (S 01º18’, W 77º52’) / 800-1200m Petr Baňař lgt. [printed]; Coll Petr Baňař / petrbanar@seznam.cz [printed] (MMBC).

1. ***Sirthenea stria* (Fabricius, 1794)**

**Brasil:** Male: Sirthenea / rosea / H. She. [handwritten]; Brasil [handwritten]; Mus. Zool. Helsinki / Loan No. / HE [printed] 5523 [handwritten]; Sirthenea / stria stria (Fabr.) [handwritten]; Det. L. Willemse 1982 [printed; date partially handwritten] (MZH). **Colombia**: Male: Col. Valle Del Cauca / Buenaventura env. / SARAGOSA 1000m / 22-28.6.1996 / V;ad/ MALY lgt. Col.3 [printed ]; Sammlung-Collection / Ernst Heiss . Innsbruck – Austria [printed]; Colombia / (neotrop.) [handwritten] (TKMF). **Costa Rica**: Female: Cordillera de Talamanca / Corina 2/2000 / Costa Rica / David Sbvoboda lgt. (MMBC). **Paraguay**: Male: Paraguay [printed]; Molinus / 10.1925 [handwritten]; F. Schade [printed]; Mus. / Zool. Helsinki / N:o [printed]; Sirthenea / suturalis / Horvath [handwritten] / JMaldonado [printed] / -1967 [handwritten]; Mus. Zool. Helsinki / Loan No. / HE [printed] 5524 [handwritten]; Sirthenea / stria stria (F.) [handwritten]; Det. L. Willemse 1982 [printed; date partially handwritten] (MZH).

1. ***Sirthenea venezolana* Maldonado, 1955**

**Ecquador**: Male: Ecquador, Napo / Prov. Limoncocha / 8 June 1977 / Dave L. Vincent [printed] (USNM)

1. ***Sirthenea vidua* Horváth, 1909**

**Costa Rica**: Female: Carpintera / Costa Rica / Reimoser [printed] (NHMW).

1. ***Sirthenea vittata* Distant, 1902**

**Panama**: Female: Tocumen R.P. / 26-I-53 / F.S Blanton [handwritten]; Museo de la Plata [printed] / Sirthenea / vittata / Dist. [handwritten] / Det. Coscarón [handwritten] (NMEG). Male: Panama / Tocumen / 25 III 1952 [printed]; FSBlanton / Collector [printed] (NMEG). Male: Tabernilla / Canal Zone / Panama [printed]; May / 04 [handwritten]; Aug. Bucsk / collector [printed]; Sirthenea / vittata / Dist. [handwritten] / det H G Barber [printed] (NMEG).

1. ***Androclus granulatus* Stål, 1863**

**India**: Male: S-India, , Tamil Nadu state, / Nilgiri hills 15 km SE of Kotagiri, Kunjappanai env., 76º56’E / 11º22’N, ca 900 m, 22-30.v.1999, / Z. Kejval & M. Tr$ý$zna leg. [printed] (MMBC).

1. ***Calistocoris virgo* Reuter, 1881**

**Malaysia**: Male: Malaysia, Pahang Distr. / 22.4-15.5.2002 / 30 Km NE of Raub [printed]; Lata Lembik env. / 3º56’N, 101º38’E, / E. Jendek et O. Šauša lgt. [printed]; Ex Collectio / Z. Jindra, Prague [printed]; Calistocoris / sp. [handwritten] / det. Rédei D., 2014 [printed] (ZJPC). Male: Indonesia. W Sumatra / May 2006, Mt. Singgalang / Annai Valley env., 600 m / S. Jakl lgt. [printed]; Ex Collectio / Z. Jindra, Prague [printed] (ZJPC).

1. ***Ectomocoris ululans* (Rossi, 1807)**

Female: Georgia / 41º17’3.43”N 45º54’11.39”E / Dalis Hotel, at light / env. of Dalis Mt Reservoir / 24.06.2017 a=380m / Roland Dobosz leg. [printed]; 5977 / 26573 / coll. Upper Silesian Museum / (USMB) Bytom, Poland [printed] (USMB) Female: Georgia / 41º17’3.43”N 45º54’11.39”E / Dalis Hotel, at light / env. of Dalis Mt Reservoir / 24.06.2017 a=380m / Roland Dobosz leg. [printed]; 5977 / 26565 / coll. Upper Silesian Museum / (USMB) Bytom, Poland [printed] (USMB) Female: Georgia / 41º17’3.43”N 45º54’11.39”E / Dalis Hotel, at light / env. of Dalis Mt Reservoir / 24.06.2017 a=380m / Roland Dobosz leg. [printed]; 5977 / 25706 / coll. Upper Silesian Museum / (USMB) Bytom, Poland [printed] (USMB). Male: Female: Georgia / 41º17’3.43”N 45º54’11.39”E / Dalis Hotel, at light / env. of Dalis Mt Reservoir / 24.06.2017 a=380m / Roland Dobosz leg. [printed]; 5977 / 26590 / coll. Upper Silesian Museum / (USMB) Bytom, Poland [printed] (USMB). Male: Female: Georgia / 41º17’3.43”N 45º54’11.39”E / Dalis Hotel, at light / env. of Dalis Mt Reservoir / 24.06.2017 a=380m / Roland Dobosz leg. [printed]; 5977 / 26540 / coll. Upper Silesian Museum / (USMB) Bytom, Poland [printed] (USMB). Male: Female: Georgia / 41º17’3.43”N 45º54’11.39”E / Dalis Hotel, at light / env. of Dalis Mt Reservoir / 24.06.2017 a=380m / Roland Dobosz leg. [printed]; 5977 / 26554 / coll. Upper Silesian Museum / (USMB) Bytom, Poland [printed] (USMB).

1. ***Peirates strepitans* Rambur, 1839**

**Republic of South Africa**: Male: Coll. I.R.Sc.N.B. / RSA, W. Cape / NE Knysna S of / Prince Alfreds pass / 24-XII-2007/G.31.175 / Lgt. M. Snižek [printed on blue label] (RBINS).**Yemen**: Female: Yemen-E, Hawf NE / Albhaydah, 200-730 m / N16º40’, E53º05’. 14. / J. Halada lg., X.2005 [printed]; Collectio / Petr Baňař / Moravian Museum Brno [printed] (MMBC).

1. **Platymeris rhadamanthus Gerstaecker, 1873**

**Angola:** Male: ~~IFAN~~ 1948 [printed, partially handwritten] / Dundo X / Angola [handwritten]; IFAN 1948 [printed, date partially handwritten] / A de Barros / Machado [handwritten] (MNHN). **Gabon**: Male: Gabon [handwritten on old label]; Platymeris / Rhadamanthus / Gerst. [handwritten] / R. Jeannel det. [printed; old label] (MNHN). Female: Gabon [handwritten on old label] (MNHN). **Democratic Republic of Congo**: Male: Museum Paris / Congo / Dybowski 128-96 [printed on old label] (MNHN). Male: Congo belge: P.N.U. / Kaziba (1140 m.) / 7-12-II-1948 / Mis. G. F. de Witte. 1258a [printed] (MNHN). Male: Congo belge P.N.U. / Mbawe (585 m.) / 17-27-XII-1948 / Mis. G.F. de Witte. 2133a [printed]; Platymeris / rhadamanthus / Gerst [handwritten] / A. Villiers det., 1951 [printed, date partially handwritten] (MNHN). **Moçambique**: Female: Museum Paris / Moçambique / Vallée Pongoué / Guengère / G. Vasse 1906 [printed on blue label]; Septembre [printed] (MNHN). Female: Afrique Orient. Anglaise / Pori: Mbuyuni / Alluaud & Jeannel / Mars 1912 – 1110m – St. 63 [printed]; Platymeris / Rhadamanthus / Gerst. [handwritten] / R. Jeannel det. [printed] (MNHN). **Senegal**: Male: Senegal [printed] / Alayes / Mizajahk / 20-01-82 [handwritten] / B. Sigwalt leg. [printed]; det. C. Weirauch [printed] 10/2000 [handwritten] / Platymeris sp. [handwritten] (MNHN). **Tanzania**: Male: Zanzibar / R.P. Le Roy [printed on old label]; Museum Paris / Oberthür 101-96 [printed on old blue label] (MNHN). Male: Museum Paris / Tanganyika (Mpala) / Oberthür 99-96 [Printed on old label]; Platymeris / Rhadamanthus / Gers [handwritten] / J. Lhoste det [printed, old label] (MNHN). Female: Zanzibar [printed on blue label]; Museum Paris / Ern. André 1898 [printed on blue label] (MNHN). Female: Tanganjika / Njassa-See, Mango / leg. Sr. Fischer [printed] (MNHN). Male: V-I-1948 [partially handwritten] / Oundo [handwritten]; V-I-1948 [partially handwritten] / Angola. A. / De Barros- / Machado. [handwritten]; Rhadamantus [handwritten] (MNHN).
