# Supplementary material for: Genetic data of museum specimens allow for inferring evolutionary history of the cosmopolitan genus Sirthenea (Heteroptera: Reduviidae)
Source: PeerJ. 2019 Apr 10;7:e6640. doi: 10.7717/peerj.6640 (PMC6462186; doi:10.7717/peerj.6640)
Supplement: Supplemental Information 2 [file peerj-07-6640-s002.docx]

| **No.** | **Isolate** | **Species** | **Year of sampling** | **Collection** | **Sex** | **Country** | **COI** | **Reference** | **18S** | **Reference** |
| --- | --- | --- | --- | --- | --- | --- | --- | --- | --- | --- |
| 1 | Sf16 | *Sirthenea flavipes* | 2005 | ZJPC | Female | India | KX258666 | Chłond et al. 2017 | MH925974 | This study |
| 2 | ZM5 | *Sirthenea stria* | 2006 | NMEG | female | Bolivia | MH894352 | This study | MH925975 | This study |
| 3 | ZM6 | *Calistocoris virgo* | 2002 | ZJPC | male | Malaysia | MH894353 | This study | MH925976 | This study |
| 4 | ZM12 | *Sirthenea dimidiata* | 2007 | MMBC | male | Laos | MH894355 | This study | MH925978 | This study |
| 5 | ZM13 | *Sirthenea nigra* | 2007 | MMBC | male | Laos | MH894356 | This study | MH925979 | This study |
| 6 | ZM14 | *Sirthenea obscura* | 1965 | NHMUK | male | Australia | MH894357 | This study | MH925980 | This study |
| 7 | ZM15 | *Sirthenea setosa* | 1981 | MHMUK | male | Malaysia | MH894358 | This study | MH925981 | This study |
| 8 | ZM19 | *Sirthenea nitida* | 1999 | MMBC | male | Laos | MH894360 | This study | MH925983 | This study |
| 9 | ZM22 | *Peirates strepitans* | 2005 | MMBC | female | Yemen | MH894362 | This study | MH925985 | This study |
| 10 | ZM24 | *Androclus granulatus* | 1999 | MMBC | male | India | MH894363 | This study | MH925986 | This study |
| 11 | ZM25 | *Sirthenea africana* | 1969 | HNMH | female | Ghana | MH894364 | This study | MH925987 | This study |
| 12 | ZM26 | *Sirthenea flaviceps* | 2011 | MMBC | male | Madagascar | MH894365 | This study | MH925988 | This study |
| 13 | ZM27 | *Sirthenea picescens* | 1988 | USNM | male | Madagascar | MH894366 | This study | MH925989 | This study |
| 14 | ZM28 | *Sirthenea rodhaini* | 1972 | NHMUK | male | Angola | MH894367 | This study | MH925990 | This study |
| 15 | ZM29 | *Sirthenea caiana* | 1996 | TLMF | male | Vietnam | MH894368 | This study | MH925991 | This study |
| 16 | ZM30 | *Sirthenea kali* | 1898 | MNHN | female | India | MH894369 | This study | MH925992 | This study |
| 17 | ZM31 | *Sirthenea nigronitens* | 1939 | RMNH | female | New Guinea | MH894370 | This study | MH925993 | This study |
| 18 | ZM32 | *Sirthenea laevicollis* | 1924 | NHMUK | female | Australia | MH894371 | This study | MH925994 | This study |
| 19 | ZM33 | *Sirthenea atra* | 1934 | USNM | female | Paraguay | MH894372 | This study | MH925995 | This study |
| 20 | ZM34 | *Sirthenea pedestris* | 1940 | USMB | male | Brasil | MH894373 | This study | MH925996 | This study |
| 21 | ZM35 | *Sirthenea peruviana* | 1977 | USNM | male | Ecuador | MH894374 | This study | MH925997 | This study |
| 22 | ZM36 | *Sirthenea vidua* |  | NHMW | female | Costa Rica | MH894375 | This study | MH925998 | This study |
| 23 | ZM37 | *Sirthenea vittata* | 1953 | NMEG | female | Panama | MH894376 | This study | MH925999 | This study |
| 24 | ZM39 | *Sirthenea ferdinandi* | 1958 | MLPA | male | Bolivia | MH894377 | This study | MH926000 | This study |
| 25 | ZM40 | *Sirthenea plagiata* | 1977 | USNM | male | Ecuador | MH894378 | This study | MH926001 | This study |
| 26 | ZM41 | *Sirthenea amazona* | 2004 | MLPA | male | Peru | MH894379 | This study | MH926002 | This study |
| 27 | ZM42 | *Sirthenea jamaicensis* | 1982 | MHMUK | female | Jamaica | MH894380 | This study | MH926003 | This study |
| 28 | ZM43 | *Sirthenea dubia* | 1925 | MACN | female | Argentina | MH894381 | This study | MH926004 | This study |
| 29 | ZM44 | *Sirthenea ocularis* | 1992 | NMEG | male | Bolivia | MH894382 | This study | MH926005 | This study |
| 30 | ZM45 | *Sirthenea venezolana* | 1977 | USNM | male | Ecuador | MH894383 | This study | MH926006 | This study |
| 31 | ZM49 | *Ectomocoris ululans* | 2017 | USMB | female | Georgia | MH894387 | This study | MH926010 | This study |
| 32 | ZM58 | *Platymeris rhadamanthus* | 2018 | DZUS | female |  | MH894388 | This study | MH926011 | This study |
| 33 |  | *Macrocephalus dorannae* |  | | | | KY501008 | Masonick et al. 2017 | GU188463 | Weirauch et al. 2011 |
| 34 |  | *Phymata fortificata* |  | | | | KY501010 | Masonick et al. 2017 | FJ230467 | Weirauch & Munro 2009 |
| 35 |  | *Neocentrocnemis stali* |  | | | | KY501003 | Masonick et al. 2017 | GU188459 | Weirauch et al. 2011 |
| 36 |  | *Glossopelta acuta* |  | | | | KY501006 | Masonick et al. 2017 | - |  |
| 37 |  | *Ptilocnemus femoralis* |  | | | | - |  | FJ230509 | Weirauch & Munro 2009 |
| 38 |  | *Ptilocerus sp.* |  | | | | - |  | GU188460 | Weirauch et al. 2011 |
| 39 |  | *Themonocoris sp.* |  | | | | - |  | GU188465 | Weirauch et al. 2011 |
